# Book Reviews

**Published:** 1895-01

**Authors:** 


### Book Reviews.

YOUNG'S ORTHOPEDIC SURGERY. A Manual of Orthopedic Surgery for Students and Practitioners. By JAMES K. YOUNG, M. D., Instructor in Orthopedic Surgery, University of Pennsylvania, Philadelphia. In one octavo volume of 446 pages, with 285 illustrations. Cloth, $4.00 ; leather, $5.00. Philadelphia: Lea Brothers & Co. 1894.

There is no more fascinating department of surgery than that which has to deal with deformities of the human body. Its attractiveness is due in part to the fact that results are often so satisfactory, and in other part because when a surgeon has been instrumental in wresting a dependent and deformed person from suffering and helplessness and restored him to usefulness and self-support, he feels that he has contributed something to the sum of human happiness.

This author is one of the younger orthopedic surgeons who has not appeared before in this rôle, hence his work will challenge criticism, perhaps, to an unusual degree. Happily, however, it will stand the test! Young begins his work in a straightforward manner, wasting but few words in an introduction, gives a brief classification of the subject and then starts out to discuss its most important subdivision—namely, Pott's disease of the spine. The author's pathology is that which is accepted by all orthopedic surgeons of the present day; his symptomatology is clear and well classified and his treatment the most modern. He gives all the varieties of jackets, corsets and supports, both in illustration and description, and altogether writes a most satisfactory chapter on this trying disease.

The next most important malady is hip-joint disease. Young's definition of this is direct, positive and acceptable. He says hip-joint disease is a chronic tubercular lesion of the coxo-femoral articulation, beginning usually as an osteitis or synovitis and terminating in recovery, ankylosis or complete destruction of the joint. There is no misunderstanding this. The tubercular origin of this disease is well established. The author's description of the various methods of treatment applicable to the several stages is most satisfactory, and is well illustrated by numerous drawings and photographs. An interesting fact connected with the study of this subject is the observation of Bruns, quoted in another place, who states that 6 per cent. of patients under ten years of age who have been cured of hip disease, 9 per cent. of those between ten and twenty years, and 7 per cent. of those above twenty years of age succumb, eventually, to tuberculosis of other organs.

The next subject of importance is knee-joint disease, the white swelling of the older authors, but the chronic tubercular lesion of the present day. This is well described and admirably treated.

We come next in the order of importance to lateral curvature of the spine. This is a much more frequent affection than the average observer would believe, owing to the fact that deformities of this class are so easily concealed by a proper adjustment of the clothing. This is essentially a disease of the female sex, but in very young children the number of the two sexes affected is about equal. It is, by far, the most common of all orthopedic affections, and every general practitioner of medicine should familiarize himself with its diagnosis and management.

The infantile palsies are next dealt with in much detail, then rickets, knock-knee, bow legs and similar conditions are considered. Club-foot, one of the most important subjects connected with orthopedics, receives most painstaking treatment, over eighty pages being devoted to its consideration. Here is another study for the general practitioner, since it is he who sees these cases first and must be armed with the most practical advice, and must not hesitate to give it. It is a reflection on surgical science to see an adult in these days hobbling along the street with club-foot.

Many of the minor subjects dealt with in this useful treatise we have not touched upon, not because of their unimportance, but on account of limited space.

As a final remark we desire to state that this is one of the most useful of recent works in this department of surgery. It is essentially a work for the general practitioner, to remind him of his duties in the way of early diagnosis in deformities, and that he is to seek, early, the aid of a skilful orthopedic surgeon.

The illustrations in the volume are exceptionally fine and its mechanical execution is satisfactory in every detail.

---

A Hand-Book of Medical Microscopy, for Students and General Practitioners, including chapters on Bacteriology, Neoplasms and Urinary Examinations. By James E. Reeves, M. D., author of A Practical Treatise on Enteric or Typhoid Fever. With a glossary and numerous illustrations (partly in colors). Price. $2.50, net. Pp. xv.—237. Philadelphia: P. Blakiston, Son & Co., 1012 Walnut street. 1894.

This little volume has been written from the standpoint of a general practitioner of more than forty years' active experience, who thinks he is well acquainted with the needs of his professional brethren. In the first chapter of this work the author shows the absolute need of every physician to possess a microscope and a good practical knowledge of microscopy, by citing cases in his practice where the diagnosis was only able to be made through a microscopical examination. The scope of the work is almost too broad for the original purpose, embracing as it does the technique of microscopy, histology, pathology, bacteriology, examination of drinking water and the like. The subjects treated are concisely and clearly written and help to introduce the beginner to the more elaborate treatises on the microscopical sciences.

The author wisely says, in his opening chapter, the time has now come when all progressive physicians and surgeons, general practitioners and specialists alike, must either themselves possess sufficient skill in microscopic technique or else be able to command the ready service of some accomplished microscopist and pathologist to do such necessary work for them.

The work teems with nicely executed illustrations and is an excellent book of its kind. W. C. K.

---

FLINT'S PRACTICE OF MEDICINE. A Treatise on the Principles and Practice of Medicine. Designed for the use of Students and Practitioners of Medicine. By AUSTIN FLINT, M. D., LL. D., Professor of the Principles and Practice of Medicine and of Clinical Medicine in Bellevue Hospital Medical College, N. Y. New (seventh) edition, thoroughly revised by Frederick P. Henry, M. D., Professor of the Principles and Practice of Medicine in the Woman's Medical College of Pennsylvania, Philadelphia. In one octavo volume of 1143 pages, with illustrations. Cloth, $5.00 ; leather, $6.00. Philadelphia : Lea Brothers & Co. 1894.

Flint's Practice of Medicine has been known to the profession for nearly thirty years, and has been used as a text-book in most medical colleges for the greater part of this period. To supply the demand, six editions have already been exhausted, hence it is a treatise that needs no introduction to physicians.

Since the death of the distinguished author, eight years ago, many changes have taken place, especially in reference to the teaching of medicine, and it is one of the peculiarities of the present time that the novitiate will be satisfied with nothing that is not the latest, particularly with reference to text-books.

In reproducing such a work after so long an interval, it is important to incorporate such changes as have taken place in order to bring the work down to the present date so that it may compete with such text-books as have been lately issued.

To secure a proper editor to superintend such a revision was no mean task, and it is fortunate that the services of Dr. Henry could be obtained. That he is a clinical investigator, writer and teacher, fully equipped to do justice to his undertaking, is shown by his record in revising this edition of Flint's Practice. In its preparation he has omitted the section on general pathology, which is in keeping with modern works on practice. Separate chairs are now allotted to special and general pathology, and teachers in this important department publish separate treatises, hence the omission of this subject from works on practice is to be expected. In this case, the book is not materially reduced in size, for the editor has contributed nearly one hundred pages of new matter, comprising more than twenty distinct articles, besides numerous interpolations in every section of the book.

Dr. Henry's editions are, in the main, such as we might have expected from the author himself, had he been living. He is especially strong in reference to diseases of the stomach, where he has introduced a radical departure from the methods of the author. He has also strengthened the department of therapeutics very materially, and most of the new topics introduced by the editor show the hand of a master.

It is probable that Flint's Practice will continue to enjoy the favor of both undergraduates and physicians for many years. It is the one work on the subject that has the right to be called classical, and so long as it keeps abreast of the progress of events, by new editions so ably edited as this one by Dr. Henry, there is no danger of its losing its place in the front rank of modern medical science.

---

TRANSACTIONS OF THE MEDICAL SOCIETY OF THE STATE OF NEW YORK. For the year 1894. Octavo, pp. 507. Published by the Society. 1894.

The annual volumes of the transactions of this society are eagerly sought by the members and by many others interested in the work of this ancient and honorable organization. The present volume is replete with interesting medical and surgical papers. The president, Dr. Herman Bendell, of Albany, chose for the subject of his anniversary address The Physician of Sacred History. This is a classic analysis of biblical history, which shows the great scholarship of the learned president.

A discussion on abdominal surgery, participated in by several distinguished men, both within and without the state, occupies the next eighty-five pages of the book. This, alone, is worth more than the price of the volume. Another discussion, on menstruation and its abnormalities, occupies thirty-seven pages, and another on diphtheria occupies fifty more pages of the book. The remainder of the work is filled with miscellaneous contributions from various sources, together with the usual list of members, including the county medical societies. An appendix containing the laws of New York of interest to the medical profession, enacted by the Legislature of 1894, and an index close the volume, which is one of the best the society has ever issued.

---

HARE'S TEXT-BOOK OF PRACTICAL THERAPEUTICS. A Text-Book of Practical Therapeutics; with especial reference to the Application of Remedial Measures to Disease and their Employment upon a Rational Basis. By HOBART ARMORY HARE, M. D., Professor of Therapeutics and Materia Medica in the Jefferson Medical College of Philadelphia. With special chapters by Drs. G. E. de Schweinitz, Edward Martin and Barton C. Hirst. New (fourth) edition, thoroughly revised and much enlarged. In one octavo volume of 740 pages. Cloth, $3.75; leather, $4.75. Philadelphia: Lea Brothers & Co. 1894.

When a book has passed through four editions in less than four years, there is very little to say about its popularity. The medical public has already testified its appreciation of the work and no medical journal could prevent its progress if it would.

The JOURNAL has already spoken in terms of favor of the previous editions and has nothing but continued praise to offer for the one under consideration. Hare is a teacher of experience who knows how to impart knowledge to students in an impressive and lasting manner. He knows how to arrange a treatise in the most systematic manner, and with a view to the greatest economy of space and words. He knows how to keep progress with advancing science and to differentiate between the true and the false. He knows, moreover, when to stop—when his subject is finished—and to say that he has applied all these principles in the construction of this work is not to exaggerate a single fact nor to overstate the truth.

In this fourth edition the author has rewritten many of the articles on drugs, remedial measures, and diseases, and has added much practical information concerning the value of those new remedies that are really useful. The work has been made to conform to the new United States Pharmacopeia. It is equally valuable to the physician and undergraduate as an authority on therapeutics, which, in its broad sense, may be stated to be the application of therapeutical measures to the treatment of disease.

---

TRANSACTIONS OF THE TEXAS STATE MEDICAL ASSOCIATION. Twenty-sixth annual session, Austin, Texas, April 24, 25, 26 and 27, 1894.

The improvement in the make-up of this volume over its predecessors is apparent. This not only applies to the mechanical appearance of the book, but also to the material that it contains. The minutes and discussions are stenographically reported and several fine illustrations embellish the book.

Among the many valuable papers that it contains, one by Dr. J. F. Y. Paine, of Galveston, on The Present Status of Symphysiotomy, deserves special mention.

The association occupies four days with its annual meeting and disposes of an enormous amount of work. It divides itself into nine sections, which are quite as many as a national or international congress should maintain.

Though there is a general improvement in this volume in most respects, there is retrogression in one—namely, it appears in paper covers this year instead of cloth.

---

FIFTEENTH ANNUAL REPORT OF THE STATE BOARD OF HEALTH OF ILLINOIS, for the year ending December 31, 1892. With an appendix containing the report on medical education and medical colleges. Revised to December 31, 1893. Springfield, Illinois: W. H. Rokker, State Printer and Binder. 1894.

The annual volumes published by the Illinois State Board of Health possess great interest, from the fact that this institution was the first in all the states to move in the direction of higher medical education. By establishing certain standards and insisting upon them as prerequisites to the practice of medicine in the State of Illinois, it not only drove quacks and incompetent physicians out of that commonwealth, but it attracted the attention of other states that were compelled in self-defense, if not from principle, to adopt similar safeguards.

This volume contains a record of the standing of medical colleges in the United States and Canada, revised to September 30, 1893, together with the medical practice laws in force in the same for a corresponding period.

It is one of the most valuable books of reference in relation to medical education published, and every person interested in reform on this subject should obtain it.

---

Materia Medica, Pharmacology and Therapeutics. By W. Hale White, M. D., F. R. C. P., Physician to, and Lecturer on, Materia Medica and Therapeutics at Guy's Hospital, London ; Examiner in Materia Medica to the Conjoint Board of England, etc. Edited by Reynold W. Wilcox, M. A., M. D., LL. D., Professor of Clinical Medicine and Therapeutics at the New York Post-Graduate Medical School and Hospital ; Visiting Physician to St. Mark's Hospital, etc. Second American edition, thoroughly revised. Small 8vo, pp. 661. Price, $3.00. Philadelphia : P. Blakiston, Son & Company, 1012 Walnut street. 1894.

Notwithstanding the fact that many works on the subjects that this treatise embraces have appeared within the past few years, yet this one occupies a deserved place in the estimation of the American profession of medicine. It possesses the advantage of conciseness as to verbiage and completeness as to arrangement.

The American editor has done his part of the work with excellent judgment, ably seconding the author in adapting it to the necessities of medical men on this side of the Atlantic.

We regard it as one of the best reference books a student can consult, and it is also admirably adapted to the needs of the general practitioner of medicine.

It embraces the most modern ideas of materia medica and therapeutics and will easily take rank among the best of its class.

The second edition has been thoroughly revised and some of it has been entirely rewritten.

---

The Medical Register of New York, New Jersey and Connecticut. For the year commencing June 1, 1894. Published under the supervision of the New York Medico-Historical Society. John Shrady, M. D., Editor. Volume XXXII. Price, $2.10. New York : G. P. Putnam's Sons, 27 West Twenty-third street ; London, 24 Bedford street, Strand. 1894.

This register is of convenient size for reference, and will be found useful for physicians and pharmacists as well as for those interested in such commercial pursuits as bring them in relation with either of these professions.

It, however, is imperfect as to omissions and other errors relating to Buffalo, that should be corrected in the next edition.

There is too much carelessness in the preparation of directories in general, and in our own city directory in particular, which, we presume, has been consulted in the preparation of this register.

---

International Clinics. A Quarterly of Clinical Lectures on Medicine, Neurology, Pediatrics, Surgery, Genito-urinary Surgery, Gynecology, Obstetrics, Ophthalmology, Laryngology, Otology and Dermatology. By professors and lecturers in the leading medical colleges of the United States, Great Britain and Canada. Edited by Judson Daland, M. D., Philadelphia, Instructor in Clinical Medicine and Lecturer on Physical Diagnosis in the University of Pennsylvania; Assistant Physician to the University Hospital; Physician to the Philadelphia Hospital and to the Rush Hospital for Consumptives. J. Mitchell Bruce, M. D. F. R. C. P., London, England, Physician and Lecturer on Therapeutics at the Charing Cross Hospital. David W. Finlay, M. D., F. R. C. P., Aberdeen, Scotland, Professor of Practice of Medicine in the University of Aberdeen; Physician to, and Lecturer on, Clinical Medicine in the Aberdeen Royal Infirmary; Consulting Physician to the Royal Hospital for Diseases of the Chest, London. Volume II Fourth series. 1894. Royal 8vo, pp. xii.—363. Philadelphia: J. B. Lippincott Co. 1894.

The first lecture in this volume is by Professor Potain, of Paris, on The Maladies with which Typhoid Fever may be Confounded.

The Buffalo contributors are Dr. Charles G. Stockton on Anemia; Dr. Roswell Park on Reamputation of the Thigh, and Dr. M. D. Mann on Pessaries.

Among other interesting lectures we may mention one by Dr. J. Henry Carstens, of Detroit, on Vaginal Hysterectomy for Malignant Disease of the Uterus; one by Dr. John B. Hamilton, of Chicago, on Tuberculosis of the Hip-joint; and one by Dr. H. W. Longyear, of Detroit, on Vaginal Hysterectomy and Curettage of the Uterus.

A number of excellent illustrations serve to elucidate the text of several of the lectures published in this volume.

---

Essentials of the Diseases of the Ear, arranged in the form of Questions and Answers. Prepared especially for Students of Medicine and Post-Graduate Students. By E. B. Gleason, S. B., M. D., Clinical Professor of Otology, Medico-Chirurgical College, Philadelphia; Surgeon in Charge of the Nose, Throat and Ear Department of the Northern Dispensary, Philadelphia. Saunders's Question Compends, No. 24. Price, $1.00. Philadelphia: W. B. Saunders, 925 Walnut street. 1894.

The illustrations in this compend are well drawn and very clear and give substantial aid to an understanding of the text.

In studying the anatomy of the ear this book will be found valuable. So, too, with reference to the various manipulations of the otoscope and instruments connected with the treatment of diseases of the ear.

---

A MANUAL OF HUMAN PHYSIOLOGY. Prepared with Special Reference to Students of Medicine. By JOSEPH H. RAYMOND, A. M., M. D., Professor of Physiology and Hygiene in the Long Island College Hospital, and Director of Physiology in the Hoagland Laboratory. Saunders's New Aid Series. Small 8vo, pp. 382. With 102 illustrations in text and four full-page colored plates. Price, $1.25 net. Philadelphia: W. B. Saunders, 925 Walnut street. 1894.

The author of this book is an experienced teacher in physiology. Taking advantage of this experience he has condensed into a comparatively small compass the essentials of the study of human physiology and has set them forth in very acceptable form. This manual, with very little modification, would make an excellent text-book for use in our public schools.

It is quite true that under our present system of medical education there is only time for students to grasp the general principles of physiology, hence some such aid as this book furnishes becomes essential in student years.

It seems to us a great pity that more time and attention is not given to physiology in the collegiate courses. It is a department of medicine that lies at the very threshold of the healing art and should be taught equally as thoroughly as anatomy, while these two and chemistry should form the entire foundation of study to be pursued during the first year, to the exclusion of all other branches.

The method Dr. Raymond has adopted in the presentation of his subject is an excellent one, and his manual is commended to students of physiology.

---

SYLLABUS OF LECTURES ON HUMAN EMBRYOLOGY. An Introduction to the Study of Obstetrics and Gynecology. For Medical Students and Practitioners. With a Glossary of Embryological Terms. By WALTER PORTER MANTON, M. D., Professor of Clinical Gynecology and Lecturer on Obstetrics in the Detroit College of Medicine; Fellow of the Royal Microscopical Society, of the British Zoological Society, American Microscopical Society, etc., etc. Illustrated with Seventy (70) Outline Drawings and Photo-Engravings. Duodecimo, cloth, 126 pages, interleaved for adding notes and other illustrations, $1.25 net. Philadelphia: The F. A. Davis Co., Publishers, 1914 and 1916 Cherry street. 1894.

The author of this syllabus is an experienced teacher, and he has given in this work an arrangement that he has followed in his lectures during the past ten years. The object of its publication, as stated in the preface, is to furnish students and practitioners of medicine an outline of the principal facts in human embryology, leaving details and theories to be obtained in larger works and special monographs.

The book is arranged to be used in the class-room, the printed headings serving as a guide during the elaboration of the subject by the teacher, while notes of the lecture may be entered on the blank pages that are inserted. Foot notes have been added to facilitate search for further information on special subjects.

The illustrations are excellent, many of which are from specimens in the author's own collection.

We commend this as one of the best works of its kind extant.

---

THE PRINCIPLES OF BACTERIOLOGY. A Practical Manual for Students and Physicians. By A. C. ABBOTT, M. D., First Assistant, Laboratory of Hygiene, University of Pennsylvania, Philadelphia. Second edition, enlarged and thoroughly revised. With ninety-four illustrations, of which seventeen are colored. Philadelphia: Lea Brothers & Co. 1894.

In the issue of the JOURNAL for April, 1892, there appeared a review of the first edition of this work, and reference was made to its many interesting features. Its success has been demonstrated by the appearance of this second edition, which is a great improvement on the first. The text has been doubled without even going into the details of bacteriology and the illustrations trebled. One happy feature of this edition is the appearance of colored illustrations, helping to show the exact appearance of the various bacilli as they appear stained under the microscope. An appendix is also given with a list of apparatus and materials required in a beginner's laboratory. W. C. K.

---

TRANSACTIONS OF THE AMERICAN SURGICAL ASSOCIATION, Volume the Twelfth. Edited by DE FOREST WILLARD, M. D., Recorder of the Association. Philadelphia: Printed for the Association and for sale by William J. Dornan. 1894.

This volume contains the papers read at the last annual meeting of this excellent association. As usual, it is replete with interest. One of the episodes of the meeting was the presentation of a gavel made from the office chair of the late Dr. Samuel D. Gross, one of the founders of the association. The gift was made by Dr. J. Ewing Mears, the retiring president of the association, and a full-page half-tone engraving of the gavel appears in the book. Dr. W. W. Keen, of Philadelphia, presents an interesting paper on shoulder amputations, in which he reports four cases. In one of these there was amputation of the entire upper extremity, including the scapula and clavicle, and in three others there was amputation at the shoulder joint. The author presented the paper with especial reference to a discussion of the methods of controlling hemorrhage. It is an admirably prepared paper, contains nineteen illustrations, and is one of the most important contributions that has lately been made to shoulder amputations.

The book is full of instructive material that every surgeon will be interested in reading.

---

THE SYMPATHETIC NERVE. Drawn from a dissection prepared by Byron Robinson, M. D., Chicago. 1894.

The author spent five years of labor in dissecting the sympathetic nerve for this plate. He states that the dissection from which the accompanying drawing was made was on a female cadaver about thirty years of age. The body was kept in alcohol for about ten months. The completed drawing is the result of one month's labor of an artist working daily under Dr. Robinson's supervision. He claims, therefore, that this representation is as close to nature as science and art are capable of reproducing. We think the claim is justified.

---

### BOOKS RECEIVED.

A Compend of the Practice of Medicine. By David E. Hughes, M. D., Chief Resident Physician Philadelphia Hospital; Physician-in-Chief, Insane Department, Philadelphia Hospital, etc., etc. Fifth physicians' edition. Thoroughly revised and enlarged. Including a very complete section on skin diseases and a new section on mental diseases. Small 8vo; pp. viii.—568. Price, $2.50. Philadelphia: P. Blakiston, Son & Co., 1012 Walnut street. 1895.

Transactions of the Colorado State Medical Society. Twenty-fourth Annual Convention. Denver, June, 1894. Published for the society.

Obstetric Surgery. By Egbert H. Grandin, M. D., Obstetric Surgeon to the New York Maternity Hospital; Gynecologist to the French Hospital, etc.; and George W. Jarman, M. D., Obstetric Surgeon to the New York Maternity Hospital; Gynecologist to the Cancer Hospital, etc. With eighty-five illustrations in the text and fifteen full-page photographic plates. Royal octavo; pp. 220. Extra cloth, $2.50, net. Philadelphia: The F. A. Davis Co., Publishers, 1914 and 1916 Cherry street. 1894.

The Principles of Surgery and Surgical Pathology. General rules governing operations and the application of dressings. By Dr. Hermann Tillmanns, Professor in the University of Leipzig. Translated from the third German edition by John Rogers, M. D., New York, and Benjamin Tilton, M. D., New York. Edited by Lewis A. Stinson, M. D., Professor of Surgery in the University of the City of New York, Medical Department. With 441 illustrations; 8vo, pp. viii.—800. New York: D. Appleton & Company. 1894.

A System of Legal Medicine. By Allan McLane Hamilton, M. D., Consulting Physician to the Insane Asylums of New York City, etc., etc., and Lawrence Godkin, Esq., of the New York Bar. With twenty-seven collaborators. Illustrated. Volume II. Large 8vo, pp. 738. Price per volume, cloth, $5.50; full sheep, $6.50. New York: E. B. Treat, 5 Cooper Union. 1894.

Difficult Labor: A Guide to its Management, for Students and Practitioners. By G. Ernest Herman, M. B., Lond., F. R. C. P., Senior Obstetric Physician to the London Hospital; Physician to the General

Lying-in-Hospital ; President of the Obstetrical Society of London ; Examiner in Midwifery to the Royal College of Surgeons ; Late Physician to the Royal Maternity Charity. Crown octavo, 442 pages, 162 illustrations. Price, muslin, $2.25. New York : William Wood & Company. 1894.

Fourteenth Annual Report of the State Board of Health of New York. In two volumes and accompanying maps. Transmitted to the Legislature February 15, 1894. Albany : James B. Lyon, State Printer. 1894.

